# Medical Superstitions

**Published:** 1897-09

**Authors:** 


### Medical Superstitions.

FOR toothache trim your finger-nails on Friday; eat bread a mouse has nibbled, or bury a tooth in the hole of a mouse, or carry in the pocket a tooth from a soldier killed in battle, or from a murdered man. Kiss a mule. Rub the gums with the body of an ant, bee or fly, or prick them with a sharp twig from a sweet apple tree. [There are no such dental superstitions.—ED. REGISTER.]

